# Craniometric guidance for the Sylvian vein

**DOI:** 10.3389/fnins.2025.1639565

**Published:** 2025-09-08

**Authors:** Chang Heon Kim, Hyo Joon Kim, Robert Seungbok Lee

**Affiliations:** ^1^Department of Neurosurgery, Presbyterian Medical Center, Jeonju, Republic of Korea; ^2^Department of Physical Medicine and Rehabilitation, Presbyterian Medical Center, Jeonju, Republic of Korea; ^3^Global Elite Division, Yonsei University, Wonju, Republic of Korea

**Keywords:** cerebral veins, Sylvian vein, anatomic landmarks, craniometry, Sylvian circle

## Abstract

**Objectives:**

The Sylvian vein (SV) is the primary anatomical landmark on the lateral surface of the brain. For the neurosurgical approach, recognizing the SV is essential information. With the trend toward minimally invasive surgery, precise anatomical localization becomes increasingly important. Moreover, a craniometric guidance for the SV will reliably enhance neurosurgical planning and intraoperative approach. Anatomical guidance for the SV was previously proposed, and it utilized a linear bar type. However, its representation was complex and unclear. Thus, we aimed to develop a new guidance for the SV.

**Materials and methods:**

The SV of thirty-seven patients was illustrated on venous phase angiographic images. Scanned images were manually fused using Adobe Photoshop CS5. The outlines of the lateral skulls were realigned to fit together, after which venous structures were overlaid. Coronal sutures and posterior clinoid processes served as references during this realignment process. Half the length of the line connecting the external ear canal (EAC) and glabella was used to draw a circle (Sylvian circle). The Sylvian circle (SC) and the actual course of the SVs were then compared. The SV and SC distributions were measured using ImageJ (NIH).

**Results:**

Twenty-nine (79%) of the thirty-seven patients exhibited SV located within 5 mm of the SC. Five SVs were positioned above the SC, and two were placed below it. There was a total of seven cases in which the trajectory was within 5 mm of the SC and accounted for less than 80% of the path.

**Conclusion:**

The SC represents the contour of the SV more accurately than a straight line. The SC can be drawn promptly and is instinctively applicable in pre- and intra-operative neurosurgical practice.

## Introduction

The Sylvian vein (SV) is a superficial cerebral vein housed in the Sylvian fissure. The vein is a representative anatomical landmark on the lateral surface of the brain (Bisaria, 1985; Yamakami et al., 1998). It traverses the opercular area around the lateral sulcus to enter the anterior temporal lobe and sphenoid sinus. According to the branching and drainage pattern within the brain’s anatomy, it is further subdivided into sphenoparietal type (54%), emissary type, cavernous type, superior petrosal type, basal type, squamosal type, and undeveloped type (Bisaria, 1985; Bonneville et al., 1989; Ebeling et al., 1989; Knott, 1881; Oka et al., 1985; San Millan Ruiz et al., 1999; Suzuki and Matsumoto, 2000).

The SV holds an important role that can provide critical information regarding the clinical application of procedures performed in neurosurgery (Bisaria, 1985; Yamakami et al., 1998; Oka et al., 1985; San Millan Ruiz et al., 1999). With the recent trend toward minimally invasive surgery (Sangha et al., 2023; Jain et al., 2003), the precise anatomical location of the SV has become increasingly valuable. It is believed that if a craniometric guide to the SV can be established, neurosurgical planning and approach will be significantly facilitated with increased safety.

Traditionally, the Taylor-Haughton line (THL) has been used as an anatomical guide for the location of the SV (Taylor et al., 1980; Taylor and Haughton, 1900). However, the THL is a simple straight line and is not easy to draw as a landmark from a neurosurgical point of view. Furthermore, it is unclear whether this accurately represents the actual location of the vein. Therefore, the authors of this study aimed to provide a pragmatic and novel craniometric guide for the location of the SV.

## Materials and methods

We reviewed cerebral angiographic images of 37 patients (Tables 1, 2; clinical characteristics and sample size). The recruited participants had no apparent vascular disease regarding the distribution of the superior vena cava (SVC). The angiographic images of the venous phase, including those taken from the lateral aspect of the skull, were developed into films, and the course of the SV was overprinted. The films were scanned using an Epson GT-9500 scanner and digitized into tagged image file format (TIFF) images containing the actual length data.

**Table 1:** Clinical characteristics.

Gender (Male:Female)	1:1.06
Total Age (Mean±SD)	61.2 ± 10.8 yrs
Male Age (Mean±SD)	63.4 ± 8.84 yrs
Female Age (Mean±SD)	59.1 ± 12.4 yrs
Skull thickness (coronal suture criteria)	6.17 ± 1.22 mm

**Table 2:** Sample size.

Group	*N*	Mean (%)	Std Dev
Male	18	79	25.9
Female	19	86.4	20.7

These patients underwent cerebral angiography as indicated during their stroke treatment. The institutional board reviewed the angiograms retrospectively for images that met the following two criteria: (1) no underlying vascular disease was detected, and (2) the skull outline required for craniometric guidance was included in the image without cropping.

The angiography examination is performed on the left anatomical side in our clinic, and for this purpose, symmetry is not assumed. This is a standard practice within this clinical field when employing most digital subtraction angiography machines. The advantage in the asymmetric laterality--favoring the left over the right side--is the decreased likelihood of distortion. Consequently, the image on the left is slightly sharper than on the right side.

**Figure 1:**
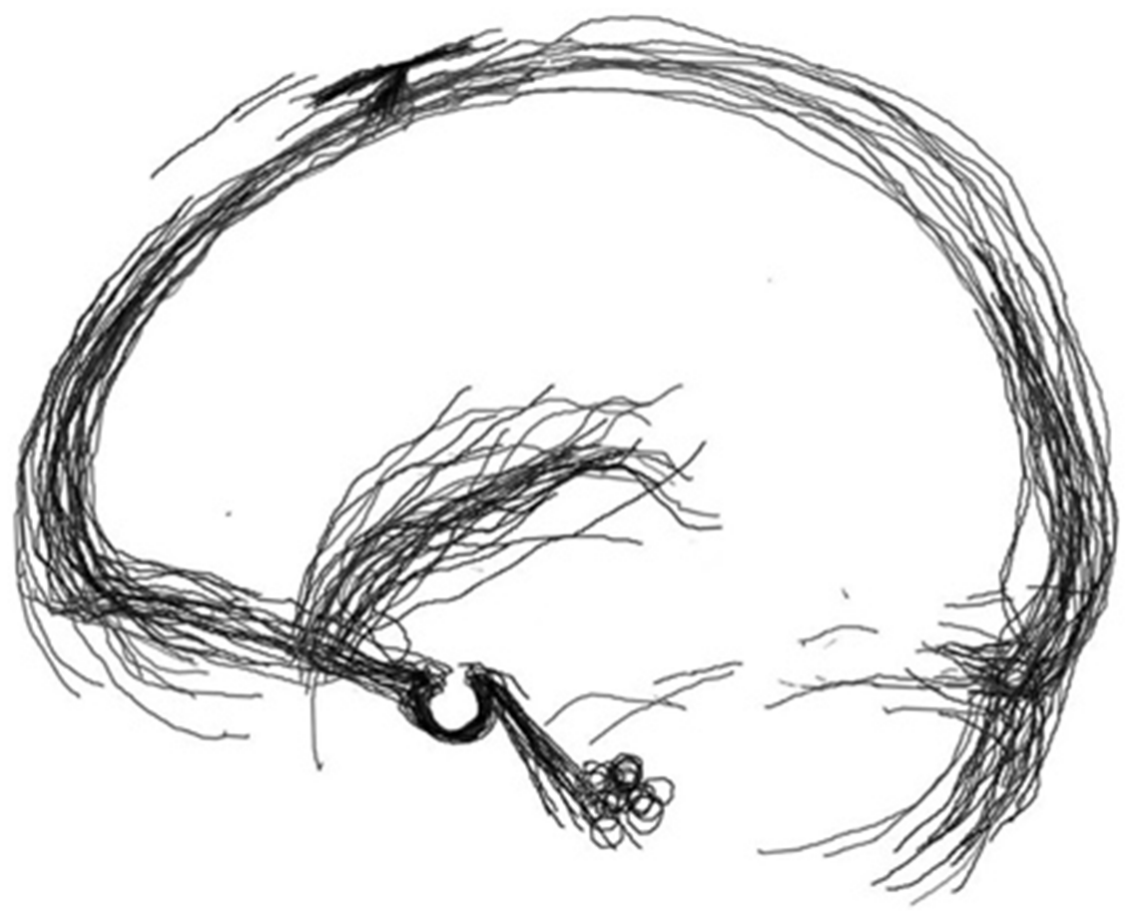
Sylvian veins were fused from cerebral angiography with two fiducials, the coronal suture, and the posterior clinoid process.

Although the SV sometimes had multiple branches, most were contiguous within the Sylvian fissure. The thickest branch was chosen to trace the path. The scanned images were overlapped, transformed, and realigned to match the size of the skull base. The coronal suture and the posterior superior process served as reference points to obtain an image showing the distribution of the SV (Figure 1) using Adobe Photoshop CS5.

**Figure 2:**
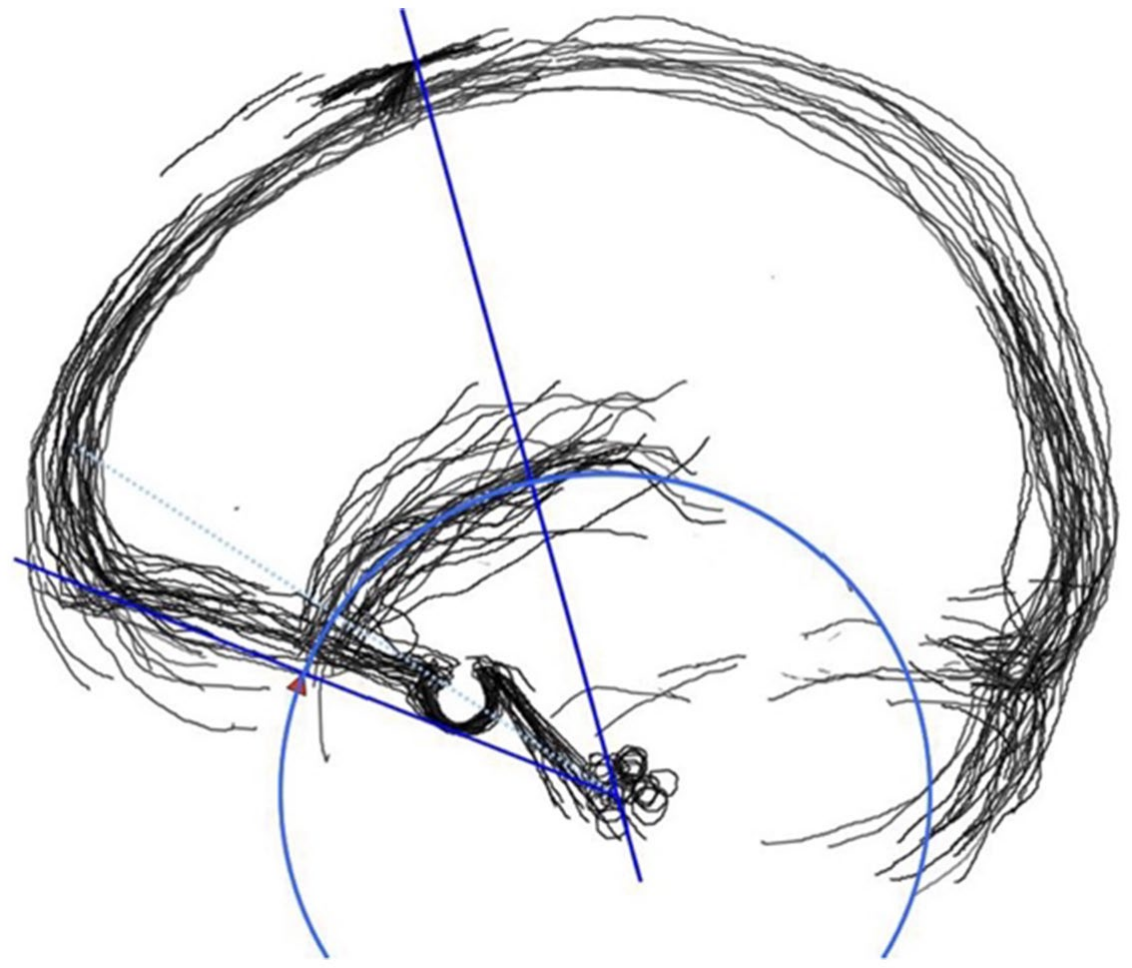
Sylvian circle (blue arc) has a radius (red arrowhead) half of the Glabella-external ear meatus line.

A virtual arc was drawn through the SV in the acquired overlapping images. Various craniometric guides were simulated using anatomical landmarks to define the arc. A straight line was drawn connecting the glabella and the external auditory canal (EAC) from the side. Then, a circle was drawn with the EAC as the axis and the midpoint between the glabella and the EAC as the radius. We found that a circle passed through the middle of the SV distribution (Figure 2). We referred to this circle as the “Sylvian circle” (SC) with the EAC as the axis and the midpoint of the line between the glabella and the EAC as the radius. Conversely, when applying the SC to each image, we checked to see if it matched the actual path of the Sylvian vein. Path concordance was determined by drawing a 5-mm-wide band inside and outside the SC. This measured the total length of the SV path and the length of the path deviating from the band.

**Figure 3:**
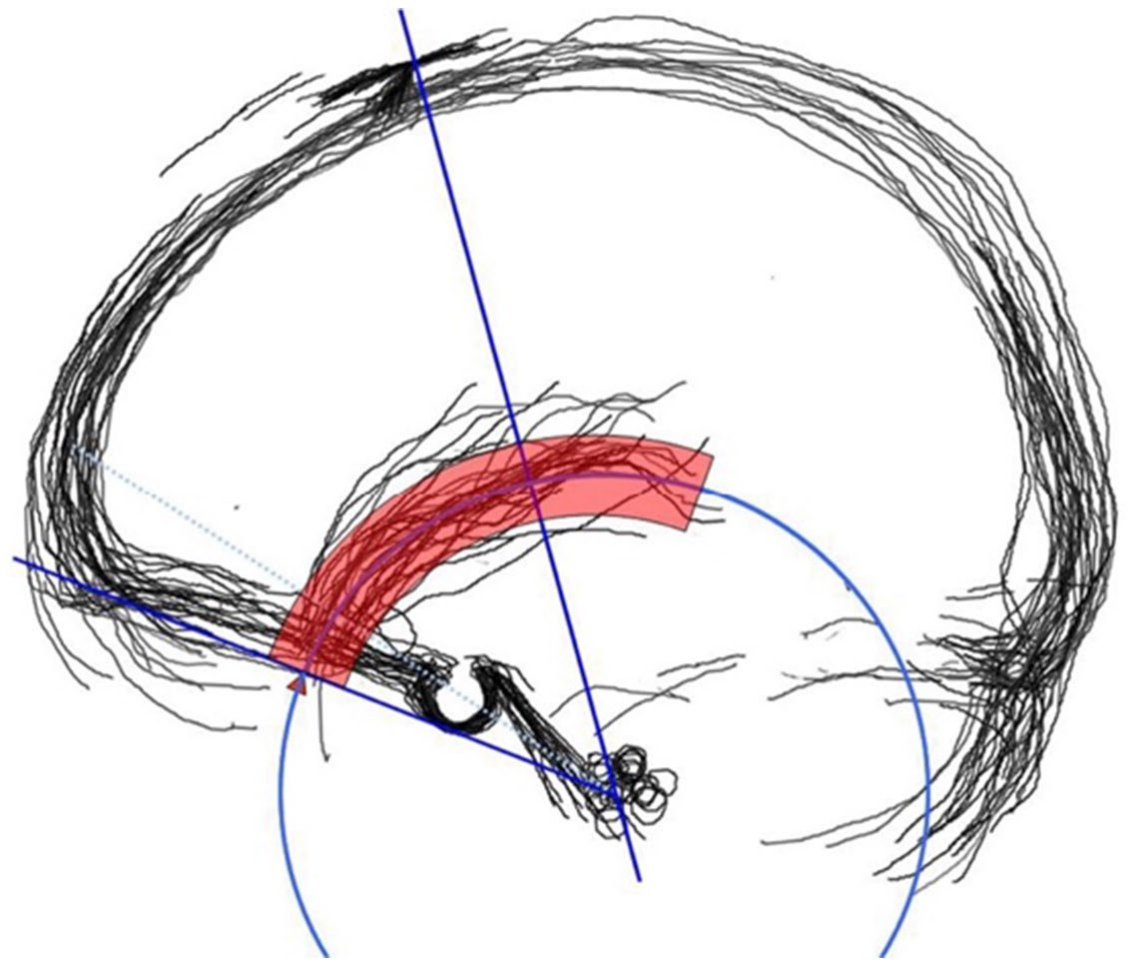
The majority of the sylvian veins run within 5 mm (red circular band) of the Sylvian circle (blue arc).

Matching was determined if the length of the deviating portion was less than 20% of the total SV length (Figure 3) (ImageJ, NIH) (Figure 3; ImageJ, NIH). In vessel path matching, “deviating portion” refers to a “segment outside of the predicted region.” It may also be labeled as an “outlying,” “deviated,” or “extraneous” segment. This application is based on previous vascular studies that demonstrated 20% to be an acceptable cutoff point for matching when considering measurement error in digital imaging (Elisevich et al., 1995; Wintermark et al., 2000; Schirmer and Malek, 2023).

**Figure 4:**
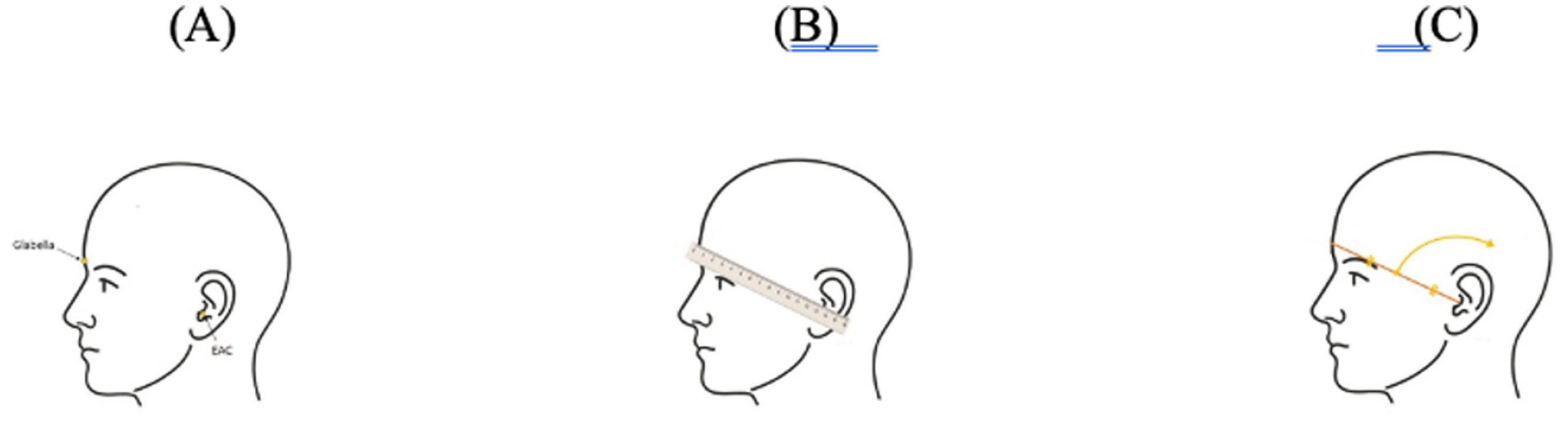
A schematic illustration of the intraoperative Sylvian circle drawing on the scalp. **(A)** The Glabella and EAC are shown. **(B)** A straight line is drawn connecting the Glabella and the EAC. **(C)** The midpoint of the Glabella–EAC line is marked.

The stepwise process of drawing the SC directly on the scalp intra-operatively and also for preoperative planning of craniotomy procedures is illustrated (Figure 4). The SC is created in the following stepwise process: (1) firstly, a straight line is drawn to connect the glabella and the EAC as shown in A and B; (2) secondly, the midpoint of the glabella–EAC line is marked as shown in C; (3) thirdly, an arc centered at the EAC is drawn, using the distance between the EAC and the midpoint of the glabella–EAC line as the radius.

## Results

The SC was obtained through various patterning attempts on images created by summing earlier angiographic images. The SC was devised as a pattern that could better represent the SV’s curvilinear course than the more straightforward craniometric guides of the past. Its reliability was evaluated based on whether the area contained the SV’s course.

The course of the saphenous vein within 5 mm of the saphenous circle accounted for 82.82% of the total (±20.16%). The course of the saphenous veins within 5 mm of the saphenous circle was consistent with the course of the saphenous veins in images from 29 patients. This represented 78.3% of the total number of enrolled patients, with an average of 91.3% (±6.55%). The course of the SV is summarized in Table 3.

**Table 3:** Course of travel by actual Sylvian vein based on Sylvian band (SC 5 mm coverage).

In range trips for total trips	82.8% (±20.16)
Number of participants whose driving route is 80% or greater in range/Total number of participants	78.3%
[Participants with a driving route within a range of not less than 80%] Length within range/length of the entire drive path	91.3%
[Participants with driving route within a range of less than 80%] Length within range/length of the entire drive path	
	52.0%

**Figure 5:**
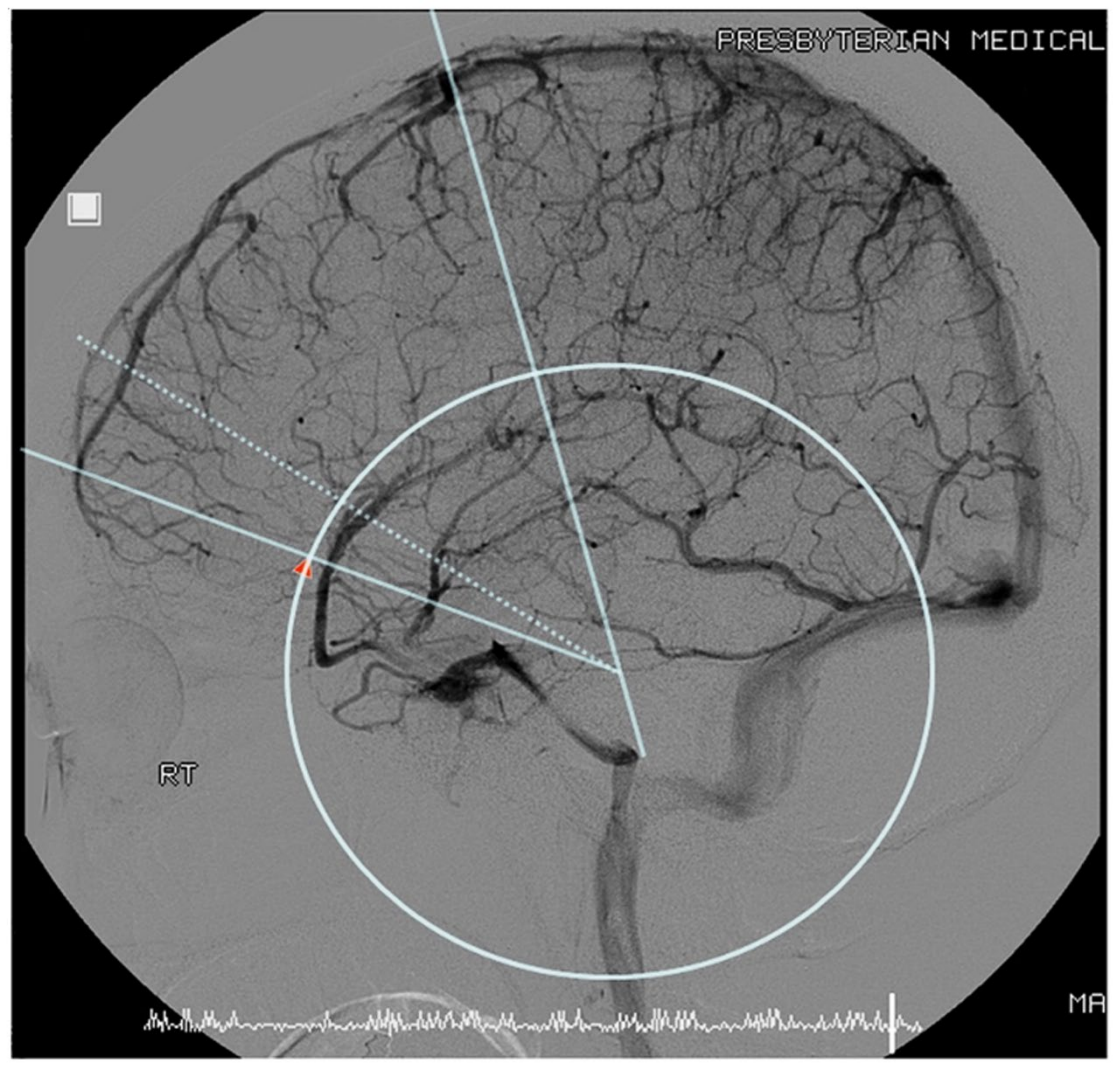
Application of the Sylvian circle to the venous phase angiography.

Of the cases that escaped the SC, five moved upward (outward) and two moved downward (inward). Photographs of the SC applied to the cranial trajectory and the SV show the SC penetrating the middle region of the trajectory dummies (Figure 5). Adjacent angiographic venous phase images demonstrate the applied SV and circle.

### Data presentation

The statistical methods used in this study are primarily descriptive. We validated the natural path of the SV and demonstrated that, like other craniometric guidelines, it accurately represents the underlying location. Statistical deficiency was addressed by comparing the agreement rate with the THL.

In each case, the farthest distance from the Sylvian line was measured to represent the distribution of the tract. Deviation from the Sylvian line is positive for the frontal side and negative for the temporal side. The y-axis is centered on the Sylvian line, and a 5 mm range is plotted to intuitively depict the up-and-down concept based on our observation.

**Figure 6:**
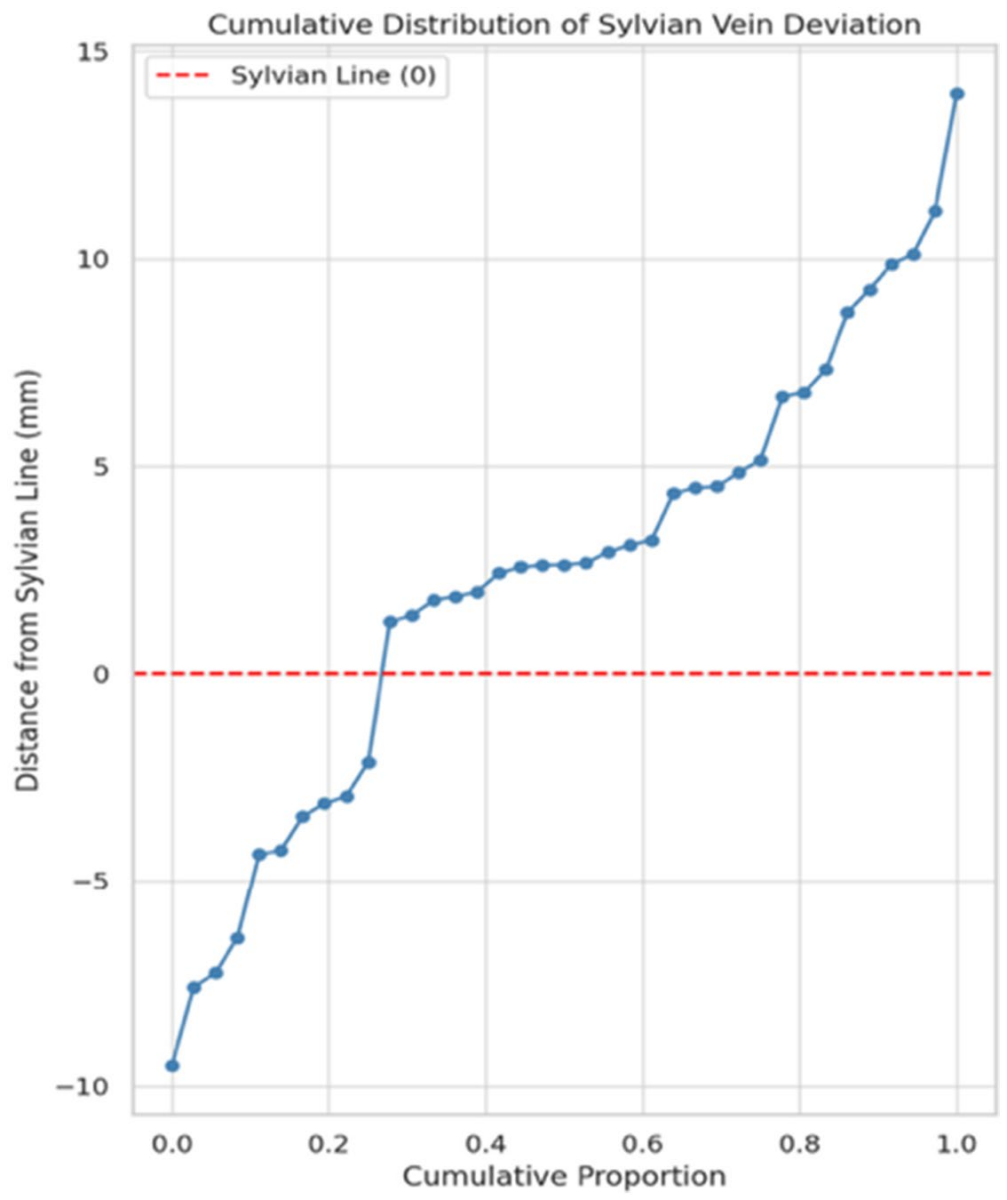
The cumulative distribution graph demonstrates the vertical distribution of Sylvian veins relative to the Sylvian line (denoted as Y = 0). Positive values represent vein trajectories located superior to the Sylvian line, while negative values indicate those positioned inferiorly.

The graph in Figure 6 depicts the deviations across all study cases (*n* = 37), emphasizing the importance of accounting for vertical dispersion when evaluating SV alignment using craniometric guides. The distribution reveals a predominance of trajectories above the Sylvian line, with a notable concentration between 0 and +10 mm. However, a subset of cases exhibited negative deviation, suggesting anatomical variability in venous path alignment. Overall, the pattern suggests a slightly superior bias, which may have surgical implications regarding access planning or risk zones.

**Figure 7:**
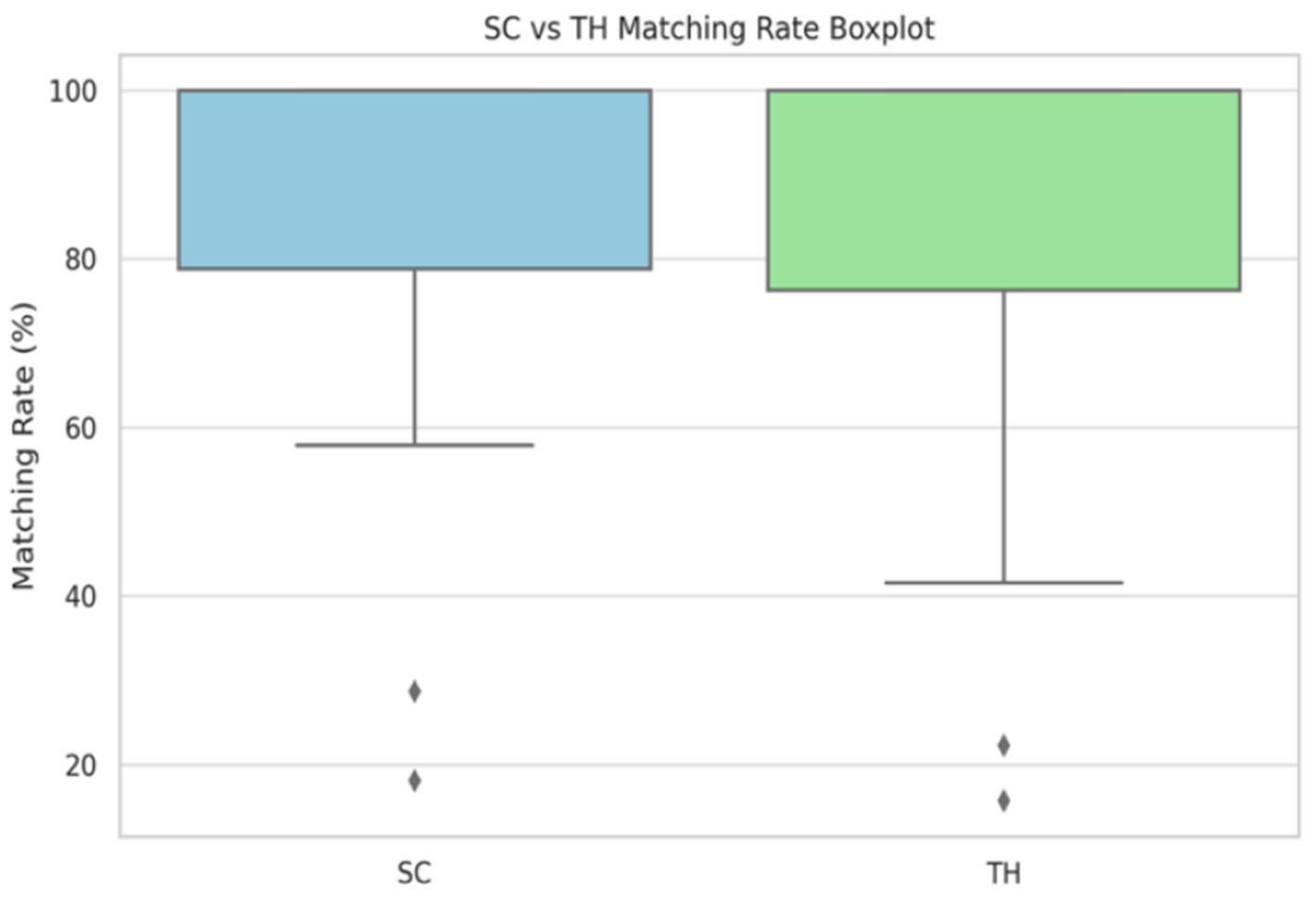
Boxplot statistics comparing the SC and the THL methods. The graph shows the distribution of matching rates for SC-based and TH-based methods. SC, Sylvian circle; TH, Talyor-Haughton line.

A Wilcoxon signed-rank test was conducted to compare the matching rates of the SC- and THL-based methods. The results revealed a statistically significant difference (W = 11.0, *p* = 0.0019) in favor of the SC-based approach. The mean matching rate was higher for the SC method (86.75%) than for the THL method (84.30%). Cliff’s Delta (*δ* = 0.053) was used to measure the effect size, which indicated a small but consistent advantage of the SC-based method. Figure 7 illustrates the distribution of matching rates for SC-based and THL-based methods using the box plot statistics. Herein, the central line indicates the median, the box represents the interquartile range (IQR), and the whiskers extend to 1.5 × IQR. Outliers are plotted as individual points.

The sample size in the study was insufficient to allow for comparisons of age and gender by age groups. Nevertheless, a comparison of SC match rates between male and female participants was conducted using a Welch’s *t*-test. The results showed no statistically significant difference between the two groups (*t* = −0.96, *p* = 0.344), suggesting that gender does not significantly affect SC matching performance.

## Discussion

The SV is an important landmark in neurosurgical planning, approaches, and procedures. Predicting its precise anatomical location can help ensure safe and accurate surgeries (Bisaria, 1985; Yamakami et al., 1998; Oka et al., 1985; San Millan Ruiz et al., 1999). In the 1900s, Taylor and Horton marked this line on the scalp at the location corresponding to the central cleft (Taylor et al., 1980; Taylor and Haughton, 1900). The line was designed to accurately mark the central cleft on computed tomography (CT) imaging (Taylor et al., 1980; Taylor and Haughton, 1900). However, its complicated composition makes applying this landmark challenging. Furthermore, it is unclear whether it accurately reflects the course of the SV.

**Figure 8:**
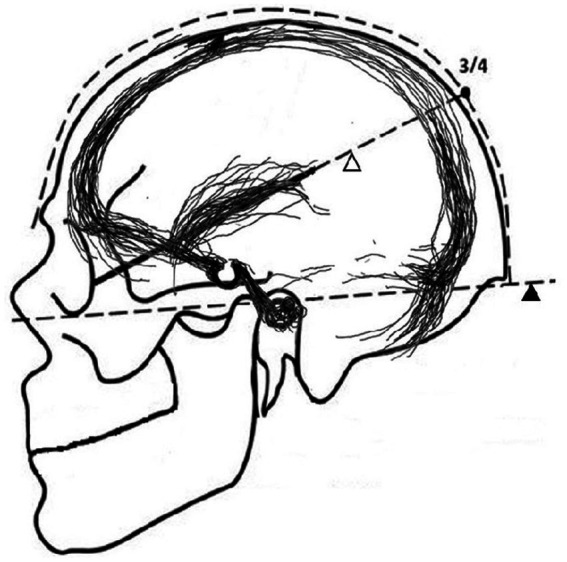
White arrowhead: Taylor-Haughton line; Black arrowhead: Frankfurt plane.

The THL (Figure 8) defines the Sylvian line that references the Frankfurt plane to represent the craniometry of the central sulcus. The Sylvian line is approximated by a line connecting the lateral canthus to the point three-quarters of the way posterior along the arc running from the nasion to the inion over the convexity. The THL indicates the presence of the SV. It is a simple, straight line derived from the location of various surrounding anatomical structures. In contrast, the SC is easy to draw using only the space between the eyebrow and the ear canal. This reliably reflects the circular shape of the SV formed by the frontal and temporal lobes.

The simple SC has numerous advantages over traditional craniometric measurements, such as the THL. Firstly, the starting point of the SC is predictable. Its purpose is to define the point of origin, which remains uncertain when using traditional methods (Figure 5). Conversely, the SC has a definitive starting point at the center of the glabella-EAC line and aligns well with the SV. Secondly, the SC is curvilinear, reflecting the curvature of the SV. This contrasts with the “straight” traditional line, representing the fundamental difference. Thirdly, the SC does not require any special equipment or instrumentation. Even without mechanical aids, such as a tape measure to mark three-quarters of the curve length, the SC can be easily drawn by hand and executed with precision. Fourthly, its simplicity offers significant benefits. The midpoint of the glabella-EAC straight line, as seen from the side, serves as the SC’s point of origin. It does not require a calculator or sophisticated instrumentation. It is simple enough for beginners and experienced professionals to use. Lastly, these advantages help the operator intuitively recognize the trajectory of the SC. The comparisons of the two anatomic landmarks—Sylvian circle and Taylor-Haughton line—are summarized (Table 4).

**Table 4:** The comparisons between SC and THL anatomic landmarks.

Category	Sylvian circle	Taylor-Haughton Line
Shape	Curvilinear, reflecting the Sylvian vein	Straight line
Ease of drawing	Simple and intuitive; can be drawn by eye without tools	Requires complex anatomical references and measurements (e.g., 3/4 arc); difficult to apply
Accuracy	Closely reflects the actual path of the Sylvian vein (82.8% within 5 mm)	Uncertain whether it accurately represents the Sylvian vein
Starting point	Clearly defined at the midpoint of the glabella–EAC (external auditory canal) line	Starting point is complex and uncertain
Practicality & accessibility	Requires no special equipment; used in any setting	Requires imaging (CT) and precise craniometric analysis
Academic value	Easily used by both beginners and experts	Difficult to apply in the surgical field
Clinical applicability	Practical in diverse surgical environments without advanced technology	May be limited to use with neuroradiologic imaging or navigation tools

The SC, as designed in the study, is formed by drawing a straight line connecting the glabella and the EAC in two dimensions from the side. The radius is halfway between the glabella and the EAC. The SC was derived by combining the SV distributions of multiple patients. When the SC was applied to the angiographic images of the patients, 82.8% of the total SV path was found to be within 5 mm of the SC.

The SV is easily visible to an experienced clinician during surgery. However, its connections with neighboring vessels are unclear in angiograms during the venous phase. The beginning and endpoints of the vasculature are also unclear, making it difficult to determine if they divide into multiple streams. Based on our intraoperative observations, we marked the area representing the SV on the film, matching its thickness and darkness. This ensures that the contour of the SV will not be lost when summarizing multiple films and removing the background besides the pattern. However, this made the clusters of thick, bold, hand-drawn lines appear rough and low quality. We could have reworked them into thin, precise lines for each film. However, since the SV has a thick, uneven contour, we chose to represent it as it is.

Therefore, this study likely requires experienced neurosurgeons or anatomists in craniotomy and venous vasculature dissection during surgery. The experienced specialists must possess the ability to distinguish the SV from other veins on lateral venous angiograms. This is step is likely the most challenging aspect of replicating the study. The fusion process vein marking was performed around the coronal suture and the Sella. Consistently distinguishing and matching the veins in angiographic images with a blurred skull could only be achieved manually since it was not possible to automate the process. Although there are software applications that can integrate and match images from the same case, we are unaware of any technology that can do so with images from different cases. Determining the contour of the SV on a venous-phase angiogram from a lateral view requires experience operating on cerebral blood vessels and may be difficult to replicate. Furthermore, insight into the newly developed configuration of the SV’s path is a unique aspect of this study and potentially presents a methodological limitation.

Other limitations are discussed as follows. While the human skull is 3D, the SC appears as a 2D construct on the scalp. This dimensional reduction can lead to minor discrepancies during CT-to-angiographic overlays or intraoperative localization. However, most scalp curvature is concentrated in the frontal and temporal regions, through which the SC predominantly traverses, remaining relatively planar. Thus, the margin of error is presumed to be minimal. Additionally, the study focused on the left side of the vasculature, where asymmetry may or may not affect applicability despite likely underlying gender differences in vascular distribution.

Another limitation is the insufficient sample size to allow comparisons of gender within age groups; the study did not differentiate possible differences in vascular distribution between genders, although these differences were summarized. However, the craniometric guidance derived from this study is clinically applicable in a gender-specific manner without detracting from the reliability of the results. Moreover, the data are based on a cohort of the Korean population, which limits generalizability. Morphometric studies have shown that, on average, Caucasians have larger intracranial volumes than East Asians, with an estimated difference of 10–15% (Dekaban and Sadowsky, 1978; Chee et al., 2011; Tang et al., 2010). However, since the present study is based on proportionally designed guidelines, the influence of ethnicity on skull morphologies or cranial size differences is expected to be minimal.

The SC is advantageous for surgical planning and execution in settings where new technologies, such as neural navigation, are challenging to use. Based on our findings, we have adopted the procedural technique in the operating room and have discovered that the SC assists in preoperative craniotomy planning when unclear boundaries of the frontal and temporal lobes are present on axial imaging. This technique helps predict the location accurately and facilitates the use of the SC approach. It helps determine anterior and posterior aspects based on the SV and assists with patient positioning and incisional precision. The use of neuro-navigation during surgery requires the use of a fixator to fix the head position. Consequently, this can affect the relationship between the SV and the focus due to head fixation. Furthermore, the distance from the scalp to the dura mater exceeds 2–3 cm. Using the tip elongation technique in neuro-navigation can result in errors of greater than 1 cm in brain surface area before craniotomy begins by using a non-fixed probe. Additionally, the SC approach is a practical and reliable method in the event of a sudden neuro-navigation device failure.

In summary, the SC is a practical and reliable landmark for preoperative planning and intraoperative neurosurgical procedures. The SC is an innovative craniometric landmark that offers a more intuitive curved guide than the traditional THL’s linear guide. It serves as an anatomical guide in establishing the basic construct of the intracranial space. The translation into a real-time intraoperative setting will inevitably add value to neurosurgical practice. Subsequent studies are warranted to achieve surgical validation of this technique. This aspect is currently pursued in our clinical setting and is in the preliminary stages of development. In the meantime, we have applied the acquired results of this study in our clinical setting. We verify that the SC is advantageously applicable in preoperative planning, especially when ambiguity of frontal and temporal boundaries on axial imaging and is intraoperatively favorable when sudden malfunction of an otherwise reliable technological device may occur.

## Conclusion

The course of the Sylvian vein is represented by the Sylvian circle, the radius of which is half the distance between the coronal suture and the external auditory canal. The Sylvian circle is a useful anatomical landmark that serves as a practical, clinically accurate, and minimally invasive craniometric guide in neurosurgical procedures. While it has a minimal impact on reducing surgery time and complications, the clinical application provides reassurance in planning and performing surgery and improves safety metrics.

## Data Availability

The original contributions presented in the study are included in the article/supplementary material, further inquiries can be directed to the corresponding author.
